# Evolutionary dynamics of polymorphic endogenous retrovirus insertions across wild house mouse populations

**DOI:** 10.1093/g3journal/jkag270

**Published:** 2026-09-22

**Authors:** Tomoki Yano, Toyoyuki Takada, Kazumichi Fujiwara, Dai Watabe, Shutaro Hirose, Hiroshi Masuya, Toshinori Endo, Naoki Osada

**Affiliations:** Graduate School of Information Science and Technology, Hokkaido University, Kita-ku, Sapporo 060-0814, Japan; Integrated Bioresource Information Division, RIKEN, BioResource Research Center, Tsukuba, Ibaraki 305-0074, Japan; Mouse Genomics Resource Laboratory, National Institute of Genetics, Mishima, Shizuoka 411-8540, Japan; Faculty of Information Science and Technology, Hokkaido University, Kita-ku, Sapporo 060-0814, Japan; School of Engineering, Hokkaido University, Kita-ku, Sapporo 060-0814, Japan; Integrated Bioresource Information Division, RIKEN, BioResource Research Center, Tsukuba, Ibaraki 305-0074, Japan; Graduate School of Information Science and Technology, Hokkaido University, Kita-ku, Sapporo 060-0814, Japan; Faculty of Information Science and Technology, Hokkaido University, Kita-ku, Sapporo 060-0814, Japan; School of Engineering, Hokkaido University, Kita-ku, Sapporo 060-0814, Japan; Graduate School of Information Science and Technology, Hokkaido University, Kita-ku, Sapporo 060-0814, Japan; Faculty of Information Science and Technology, Hokkaido University, Kita-ku, Sapporo 060-0814, Japan; School of Engineering, Hokkaido University, Kita-ku, Sapporo 060-0814, Japan

**Keywords:** endogenous retrovirus, *Mus musculus*, murine leukemia virus, adaptive introgression, *Fv4*

## Abstract

Endogenous retroviruses represent a major source of structural variation in mammalian genomes, yet their diversity in wild populations remains poorly understood. Here, we conduct a comprehensive genome-wide survey of nonreference endogenous retrovirus insertions in wild house mice (*Mus musculus*) to characterize their distribution and evolutionary dynamics. Using a newly developed, computationally efficient bioinformatics pipeline, we detected and annotated over 100,000 nonreference endogenous retrovirus insertions from short-read sequencing data across 163 wild mouse genomes sampled across Eurasia. Our analyses revealed marked differences in endogenous retrovirus insertion patterns among subspecies and populations, including variation in genomic localization and population-specific polymorphisms. These heterogeneous patterns suggest distinct evolutionary histories and host–retrovirus interactions across populations. As a case study, we describe the distribution of the endogenous retrovirus-derived *Fv4* gene, which is absent in the mouse referenece genome and confers resistance to murine leukemia viruses. Several lines of evidence showed that the spread of *Fv4* insertions in Korean population has been driven by adaptive introgression from neighboring populations. Our study provides the first large-scale population genomic scan of endogenous retrovirus diversity in wild house mice. By cataloging extensive polymorphism in nonreference endogenous retrovirus insertions, our results highlight the role of endogenous retroviruses as dynamic genomic elements that contribute to structural variation and adaptive evolution.

## Introduction

Endogenous retroviruses (ERVs) are transposable elements derived from RNA viruses that encode reverse transcriptase and become endogenized in host genomes through integration into germline DNA. ERVs are classified into 3 major classes (classes I–III) (Stoye and Coffin 1987; Stocking and Kozak 2008) and collectively comprise approximately 5% of both human and house mouse reference genomes (Lander et al. 2001; Mouse Genome Sequencing Consortium et al. 2002; Nellåker et al. 2012). The classification of ERVs is largely based on phylogenetic similarity. Class I ERVs are gammaretrovirus-like and include murine leukemia viruses (MLVs). Class II ERVs are betaretrovirus-like and are also referred to as the ERVK-like family. HERV-K, 1 of the best-studied active human ERVs, belongs to class II. Class III ERVs are spumaretrovirus-like and include many old lineages (Stoye and Coffin 1987; Stocking and Kozak 2008). ERVs can alter organismal phenotypes through multiple mechanisms, including nearby-gene silencing, insertional disruption, and regulatory activation via long terminal repeats (LTRs). In addition to these functions, several ERV or ERV-like elements have been shown to influence resistance to retroviral infection (Ikeda et al. 1981; O’Brien et al. 1983; Best et al. 1996; Dodding Mark et al. 2005; Wu et al. 2005; Zhang et al. 2008), and studies of wild house mice suggest that the distribution of ERV-derived restriction factors may reflect historical adaptation to viral pathogens (Boso et al. 2021; Fujiwara et al. 2024). Similar antiviral functions of ERVs have been raised in humans (Frank et al. 2022), yet direct evidence remains scarce. These observations highlight the need for comprehensive, population-scale surveys of ERV insertions in natural populations to understand the mechanisms by which the phenotypic variation has been shaped by originally exogenous viral components.

In laboratory house mice (*Mus musculus*), including both classical and wild-derived inbred strains, extensive variation in ERV insertion patterns has been documented (Nellåker et al. 2012; Ferraj et al. 2023), yet the genome-wide diversity of ERV insertion polymorphisms in wild populations remains unexplored. Here, we focus on nonreference polymorphic ERV insertions segregating in wild house mice. House mice comprise 3 major subspecies, *M. m. castaneus*, *M. m. domesticus*, and *M. m. musculus* (Boursot et al. 1996; Fujiwara et al. 2022), whereas classical inbred strains predominantly possess a *domesticus*-like genetic background (Keane et al. 2011). Because the reference genome of house mice derives from C57BL/6J (Mouse Genome Sequencing Consortium et al. 2002), it lacks many ERV insertions present in natural populations. Given the extensive genetic diversity among wild house mice and the presence of multiple active ERV families, characterizing nonreference insertions in wild populations provides insight into the population genetic processes shaping ERV variation. Moreover, house mice remain susceptible to active MLVs, particularly class I retroviruses such as Friend and Moloney leukemia viruses. The interplay between exogenous retroviruses and resident ERVs may therefore leave characteristic evolutionary signatures in wild house mouse genomes.

However, systematic detection of ERV insertions in nonhuman species has been hindered by both technological and analytical limitations. Long-read sequencing platforms such as Oxford Nanopore and PacBio can resolve complex structural variants (Logsdon et al. 2020; Chu et al. 2021), but sequencing large numbers of individuals with these technologies remains prohibitively expensive at present. In contrast, short-read whole-genome datasets are widely available across diverse species, yet existing ERV detection tools were largely designed for humans, often exhibit high false-discovery rates, and scale poorly with large sample sizes (Keane et al. 2013; Gardner et al. 2017; Chen and Li 2019; Bowles et al. 2023; Kojima et al. 2023). Searching for a broad spectrum of transposable elements also incurs substantial computational cost, limiting practical application in population genomic studies. To address these limitations, we developed ERVscanner, a new ERV detection pipeline optimized for high computational speed and relatively low false-discovery rates. We benchmarked ERVscanner using the inbred strain genomes with both short- and long-read sequencing data (Ferraj et al. 2023; Takada et al. 2025) and evaluated the degree of overlaps between the 2 datasets.

To demonstrate the utility of ERVscanner and to explore potential adaptive ERV insertions, we examined *Fv4*, a well-studied ERV-derived restriction factor absent from the reference genome. *Fv4* encodes an env protein derived from a class I retrovirus (Odaka et al. 1978; O’Brien et al. 1983; Inaguma et al. 1991), exhibits presence/absence polymorphism, and is most common in *M. m. castaneus*. In East Asia, secondary contact between subspecies *castaneus* and *musculus* forms a broad hybrid zone (Fujiwara et al. 2022), which can facilitate introgression of potentially adaptive alleles (Huerta-Sanchez et al. 2014). Although Korean house mice display predominantly *musculus*-like genomic backgrounds, previous studies found many individuals harbor *Fv4* (Inaguma et al. 1991; Boso et al. 2021). Our analyses indicate that *Fv4* was recently introgressed from the subspecies *castaneus* into *musculus* and that surrounding genomic patterns of polymorphisms are consistent with a scenario under selective sweep, supporting previous functional evidence for its role in retroviral resistance.

Application of the same framework revealed 3 additional ERV insertions showing population patterns compatible with selective sweep in Korean house mice. Although their functional relevance remains unclear, these loci represent promising candidates for further investigation and illustrate how scalable ERV discovery can reveal previously unrecognized variation. Our study provides a practical tool for ERV detection in short-read datasets, a population-scale catalog of ERV insertions in wild house mice, and prioritized loci, including *Fv4*, for future functional and ecological analysis.

## Materials and methods

### Development of ERVscanner

ERVscanner is a pipeline designed to detect nonreference insertions of ERV sequences using short-read mapping files (BAM or CRAM) as input. Assuming that nearly all nonreference ERV insertions have homologous sequences annotated as ERV regions in the reference genome, we can significantly reduce the computational search space. Additionally, ERVscanner avoids assembling insertion sequences, a process that is typically time-consuming, because such assemblies are often error-prone due to the nature of short-read sequences and high complexity of insertion sequences. Instead of inferring the exact sequence of insertions, ERVscanner estimates the class, family, and orientation of candidate insertions for each individual, outputting the results in VCF format. All codes are distributed at Github (https://github.com/Qtom-99/ERVscanner). ERVscanner consists of 4 main steps: filtering of ERV-associated read pairs, identification of candidate insertion loci, inference and filtering of insertion content, and genotyping of the retained loci. Detailed procedures and filtering criteria are provided in Supplementary File 1, Supplementary Table 1, and Supplementary Figs. 1 and 2.

### Analysis of JF1 mouse genome

The short-read and long-read data for the JF1 strain, which has a predominantly *musculus*-like genetic background, were obtained from Takada et al (2025). Variant data based on long-read sequencing of the JF1 strain were available from website (https://molossinus.brc.riken.jp/pub/MoGplus30data/). The short-read data (DDBJ DRA accession number: DRA006630) were randomly downsampled to 30× coverage. Because the long-read variant data were based on GRCm39, the coordinates of variants were converted to GRCm38 using the liftOver tool. To infer insertion contents from long-read sequencing data, we extracted insertion sequences ranging from 200 to 10,000 bp and performed a homology search using BLASTN (Altschul et al. 1990) against Dfam nonredundant profile HMM (NRPH) consensus sequences. For each insertion, we retained BLAST hits with an *E*-value ≤ 1 × 10^−30^, query coverage ≥50%, and an alignment length ≥200 bp, and used the hit with the highest bit score to assign the ERV family and class.

ERVcaller 1.4 was run using Dfam NRPH consensus sequences as seed sequences for insertions (Chen and Li 2019). Because we were unable to complete the analysis of the JF1 strain using the default ERVcaller parameters, we modified the code to reduce the internal parameter specified by -c in BWA-MEM (Li and Durbin 2009) for read extraction to 1/10 of the original value (from 100,000 to 10,000). This modification significantly reduced the running time but may have affected accuracy to some extent. After running ERVcaller, we extracted insertions classified as the ERV classes.

For ERVscanner analysis, we downloaded ERV sequence annotations for GRCm38 from the Dfam database. Segments classified as ERV1 and ERV2-group were considered as candidate ERV sequences. Note that the ERV2-group class includes ERV2, 3, 4, and Lentivirus families. In addition, the RLTR4_MM-int family, which is not classified as the ERV class but classified as the Long_Terminal_Repeat_Element class, was included in the analysis because a previous study classified RLTR4_MM-int as MLV-related sequences (Kawase and Ichiyanagi 2024). Likewise, the MaLR class was treated as class III. Using the information, we constructed BED and FASTA files to represent the ERV-homologous regions (masked region). To further characterize known ERV-derived restriction factors, we added the sequences of *Fv4*, *RMCF1*, and *RMCF2* in the public database (DDBJ/EMBL/Genbank accession numbers: AF490353, AF490352, AY999005, AH001845, M33884, and AH001894).

For the comparison of long-read and short-read analyses, adjacent insertion loci located within 40 bp in the long-read data were merged to avoid double-counting the same insertion due to slight differences in inferred breakpoint positions. ERVscanner-derived insertion loci were already sufficiently separated by the locus-definition procedure and therefore did not require additional merging. We then compared the 2 datasets and considered ERVscanner and long-read insertion calls to match when their inferred insertion sites were within 40 bp.

### Analysis of 11 inbred strain genomes

We retrieved insertion and deletion variant data for 20 mouse inbred strains identified using long-read sequencing technology in the study by Ferraj et al. (2023). We used the variant data called for each sample (sv_indsdel_alt_.vcf.gz) and a merged variant data (variants_freeze5_sv_indsdel_alt.vcf.gz). Short-read whole-genome sequencing data for these strains were downloaded from the project website of the Sanger Institute (https://mousegenomes.org/mouse-strains-sequenced). In total, 11 strains were shared between the 2 datasets (129S1/SvImJ, A/J, BALB/cByJ, BALB/cJ, C3H/HeJ, CAST/EiJ, DBA/2J, NOD/ShiLtJ, NZO/HlLtJ, PWK/PhJ, and WSB/EiJ) and were used for further analyses. All data were based on the GRCm39 reference genome. From the long-read VCF data, identified ERV insertion sequences were annotated using the same criteria as in the JF1 analysis. No ERV insertion calls in this dataset were located within 40 bp of one another.

Because Dfam annotations were not available for the GRCm39 reference genome, we used RepeatMasker software (Smit 2008–2015) to annotate repeat sequences in GRCm39. We constructed a nonredundant annotation, following the algorithm used in Dfam; a repeat annotation was discarded when 75% or more of its length was covered by another repeat annotation with a higher score.

ERVscanner was executed using the same parameter settings as in the JF1 analysis. Because inbred strains are highly homozygous, we encoded the presence or absence of each insertion as 1 or 0, respectively, calculated pairwise genetic distances based on these binary genotypes, and constructed neighbor-joining trees using the Bio.Phylo module in Python using the merged dataset.

### Analysis of ERV insertion polymorphisms in 163 house mice

Short-read alignment files from 163 wild *M. musculus* and 7 *Mus spretus* samples analyzed by Fujiwara et al. (2024) were used for this analysis. The dataset consisted of 136 samples sequenced using DNBSEQ at approximately 30× median coverage and 34 samples sequenced using Illumina short-read sequencing at >20× median coverage (Harr et al. 2016). These samples were collected across Eurasia, mostly with 1 or 2 individuals from each locality except for a few populations. Geographic labels were based on sampling localities, and subspecies assignments were taken from Fujiwara et al. (2024), in which each individual was assigned to 1 of the 3 subspecies based on its major genetic component inferred using ADMIXTURE (Alexander et al. 2009). Although the data were generated using different sequencing platforms and came from multiple collections, all raw reads were processed uniformly in Fujiwara et al. (2024) by mapping them to the GRCm38 reference genome. All data used for the population genetic analyses have been processed in our laboratory using the same read mapping parameters as the JF1 analysis. The resulting CRAM files were used in the present study. Details of sample collection, sequencing, read mapping, and population assignment are described in Fujiwara et al. (2024).

We used the CRAM files from the dataset to infer nonreference ERV insertions. Insertion sites were inferred using ERVscanner with the same procedure as in the JF1 analysis. Principal component analysis (PCA) using the insertion polymorphism data was performed using scikit-learn library of Python. Gene annotation of GRCm38 genome was performed using SNPEff software (Cingolani et al. 2012). The association between ERV insertion frequency and recombination was examined using the recombination rate data obtained by Liu et al. (2014).

### Analysis of 163 house mice single nucleotide polymorphism data

The same 163 wild house mice dataset was used for the population genetic analysis of *Fv*4 regions. All individual genotypes were phased using ShapeIt4 software (Delaneau et al. 2019), using the recombination rate estimated by Liu et al. (2014). Only bi-allelic single nucleotide polymorphisms (SNPs) located in nonrepetitive regions with mappability scores of 1, as estimated using GenMap software with the parameters -K 30 and -M 2 (Pockrandt et al. 2020), were retained for this dataset.

### Population genetic analysis of *Fv4* locus

For the population genetic analyses around *Fv*4, we focused on the Korean population, in which *Fv*4 was observed at high frequency. We used 11 Korean individuals as the focal population and 5 northeastern Chinese (Liaoning, Jiling, and Heilongjiang Provinces) and 10 southern Chinese individuals as comparative populations. The sample labels and population assignments are provided in Supplementary Table 2.

Population genetic summary statistics were calculated to detect the signature of selective sweep surrounding the nonreference ERV insertions. Nucleotide diversity (*π*), Tajima's *D*, and *F*_ST_ between 11 Korean and 5 northeastern Chinese samples were calculated using VCFtools (Danecek et al. 2011), for 5-kbp-length windows with step size of 1,000 bp.

For the calculation of normalized selection (nSL) and cross-population normalized selection score (XP-nSL) statistics, we assigned ancestral alleles using the genotypes of 6 *M. spretus* (SPR), and modified the VCF file using the information. The VCF files were modified so that the *M. spretus* allele was used as the REF allele (GT = 0) with genotype coding correspondingly reversed. Only the monomorphic sites in *M. spretus* were used for the analysis. Both statistics were calculated using selscan 2.0 software with default parameters (Szpiech 2024). For XP-nSL, we used Korean samples for the test population and northeastern Chinese samples for the reference population.

Local ancestry of haplotypes was assigned using RFMIX2 (Maples et al. 2013). We assumed that haplotypes surrounding *Fv4* were introgressed from southern Chinese populations (S-CHN) to northern East Asian populations. We therefore estimated the haplotype ancestry of Japanese (JPN), Korean (KOR), and northeast Chinese (NE-CHN) individuals using Kazakhstan and southern Chinese samples as reference individuals to define *musculus*- and *castaneus*-type haplotypes. The Kazakhstan population was selected as a representative population with a predominantly *musculus*-like genetic background because it showed the lowest level of admixture with the subspecies *castaneus* in a previous study (Fujiwara et al. 2022).

*f*_4_ statistics for assessing local ancestry were estimated using scikit-allel python libraries. We divided the genomic region of chr12: 75,000,000 to 85,000,000, which harbors the insertions site of *Fv*4, into nonoverlapping 20-kb-length windows and estimated *f*_4_(SPR, S-CHN; KOR, NE-CHN) for each window.

Genealogies surrounding the *Fv4* insertion site were inferred using RELATE from the full dataset of 163 individuals, following the procedure described in Fujiwara et al. (2024). In the present study, however, we re-inferred changes in population size using only chromosome 12 sequences from the subspecies *musculus* samples, with 5 rounds of iteration. The resampled genealogies were used as input for CLUES to infer the allele-frequency trajectory and selection coefficient of the *Fv4* insertion in the Korean population (Stern et al. 2019). The likelihood of the inferred selection model was compared with that of the neutral model, and the log-likelihood ratio was used to evaluate support for selection. For the CLUES analysis, 100 sets of branch lengths were resampled using RELATE. CLUES was run with default settings unless otherwise specified. We assumed a mutation rate of 5.7 × 10^−9^ per generation (Milholland et al. 2017).

### Candidate ERV insertions under adaptive evolution

To identify candidates for ERV insertions linked to selective sweep signals, we focused on loci in the Korean population showing high nSL statistics. This analysis was designed as a candidate search rather than an exhaustive genome-wide scan for all adaptive ERV insertions. SNPs in the top 0.1% of nSL scores were then pruned using PLINK with the --indep-pairwise 20 5 0.8 option to remove highly redundant SNPs within local regions. The pruning removed 91% (6,696/7,303) of the SNPs. This empirical cut-off was chosen to focus on the strongest haplotype-based signals and is comparable to, or more conservative than, outlier thresholds used in previous genome scans based on haplotype-based selection statistics (eg Pless et al. 2023; Xu et al. 2024).

After pruning, we selected SNPs that had no exons within 10 kb and were located within 1 kb of ERV insertions. This filtering was performed to identify sweep signals linked to ERV insertions rather than to nearby genes. Loci were retained if the allele frequency of the ERV insertion was greater than 0.5 in the Korean population and higher than that in subspecies *castaneus*. Because this analysis was based on a limited number of samples, we interpreted the retained loci as conservative candidates for ERV insertions linked to selective sweep signals, rather than as a comprehensive set of adaptive insertions.

## Results

### Identification of ERV insertions in JF1 mouse using long-read sequencing data

We first characterized nonreference ERV insertions in the JF1 mouse strain (Koide et al. 1998; Takada et al. 2013), identified using high-depth long-read genome sequence data (Takada et al. 2025). In total, we identified 6,558 insertions and 810 deletions, ranging from 200 to 10,000 bp and homologous to ERV sequences. As observed in previous studies, the most abundant class of insertions was class II (ERV2/ERVK), including IAP elements, comprising 77.2% of all ERV insertions. The numbers of insertions for classes I (ERV1) and III (ERV3/ERVL and ERVL-MaLR) were nearly equivalent: 10.8% for class I and 12.0% for class III. The number of deletions showed a similar trend. Among class I, the MuLV family, including internal sequences (MuLV-int), was the most abundant.

The distributions of insertion/deletion length are shown in Supplementary Fig. 3. While the majority of insertions and deletions are short, including fragmented copies and solo LTRs, 2,236 insertions and 126 deletions were in the range between 2,000 and 10,000 bp.

### Comparison between long-read and short-read datasets

In order to effectively handle large-scale population genomic data of short-read genome sequences, a new bioinformatics pipeline, ERVscanner, was developed. ERVscanner follows the general strategy used by previous mobile-element detection methods but differs in its extensive use of reference genome repeat annotations and cross-sample information. In this study, ERVscanner largely relies on the repetitive sequence annotation for the reference genome by Dfam database (Storer et al. 2021). In addition to the Dfam annotation, we can incorporate other customized seed sequences for potential insertions. In this study, we used sequences of *Fv4*, *RMCF1*, and *RMCF2*, which are known antiviral factors derived from retroviruses, as additional targets for nonreference insertions. Detailed algorithm is described in Supplementary File 1. Note that ERVscanner is not designed to investigate deletions, as identifying deletions of repetitive sequences using short-read sequencing is highly challenging.

We evaluated the computational speed, sensitivity, and concordance of ERVscanner with long-read-based insertion calls using the data from the JF1 mouse strain. ERVscanner is designed to enhance accuracy by leveraging data from multiple individuals. However, for benchmarking purposes, we evaluated the performance using only a single individual of the JF1 strain to ensure a fair comparison. In addition, the sequence depth was limited to 30-fold by downsampling the original data. For comparison, we selected ERVcaller, a tool reported for the highest sensitivity and precision in a benchmarking study using simulated datasets (Bowles et al. 2023). We note that this comparison was intended as a practical reference under fixed parameter settings rather than as a comprehensive benchmark across multiple parameter combinations.

Both pipelines analyzed all classes of ERVs using reference-mapped short-read data. Utilizing 8 cores of an Intel Xeon CPU E5-2620 v4 at 2.10 GHz, ERVscanner detected 6,177 insertions in approximately 3 h. For ERVcaller, we estimated all nonredundant transposable elements rather than only ERV sequences and extracted inferred insertions matching ERVs to enhance accuracy. Using stringent criteria (support levels 4 and 5), ERVcaller identified 11,850 candidate ERV insertions in 82 h with the same computer used for ERVscanner. All 3 methods successfully identified the insertion of an endogenous retrovirus *β*4 (ERVB4) element in the *Ednrb* gene, responsible for the characteristic piebald coat color phenotype of JF1 mice (Yamada et al. 2006; Tanave and Koide 2020).

Figure 1 presents a Venn diagram comparing the results for JF1 strain ERV insertion calls obtained from long-read sequencing, ERVscanner, and ERVcaller for the JF1 strain. Many insertions identified in the long-read call set were not detected by either short-read method, consistent with the known difficulty of detecting repetitive-element insertions from short-read data. ERVcaller identified a larger number of insertions that overlapped with the long-read call set than ERVscanner, whereas approximately 59% of the ERVcaller calls were not supported by the long-read calls. On the other hand, approximately 28% of the ERVscanner calls were not overlapped with the long-read calls, indicating higher concordance of ERVscanner with the long-read call set.

**Fig. 1.**
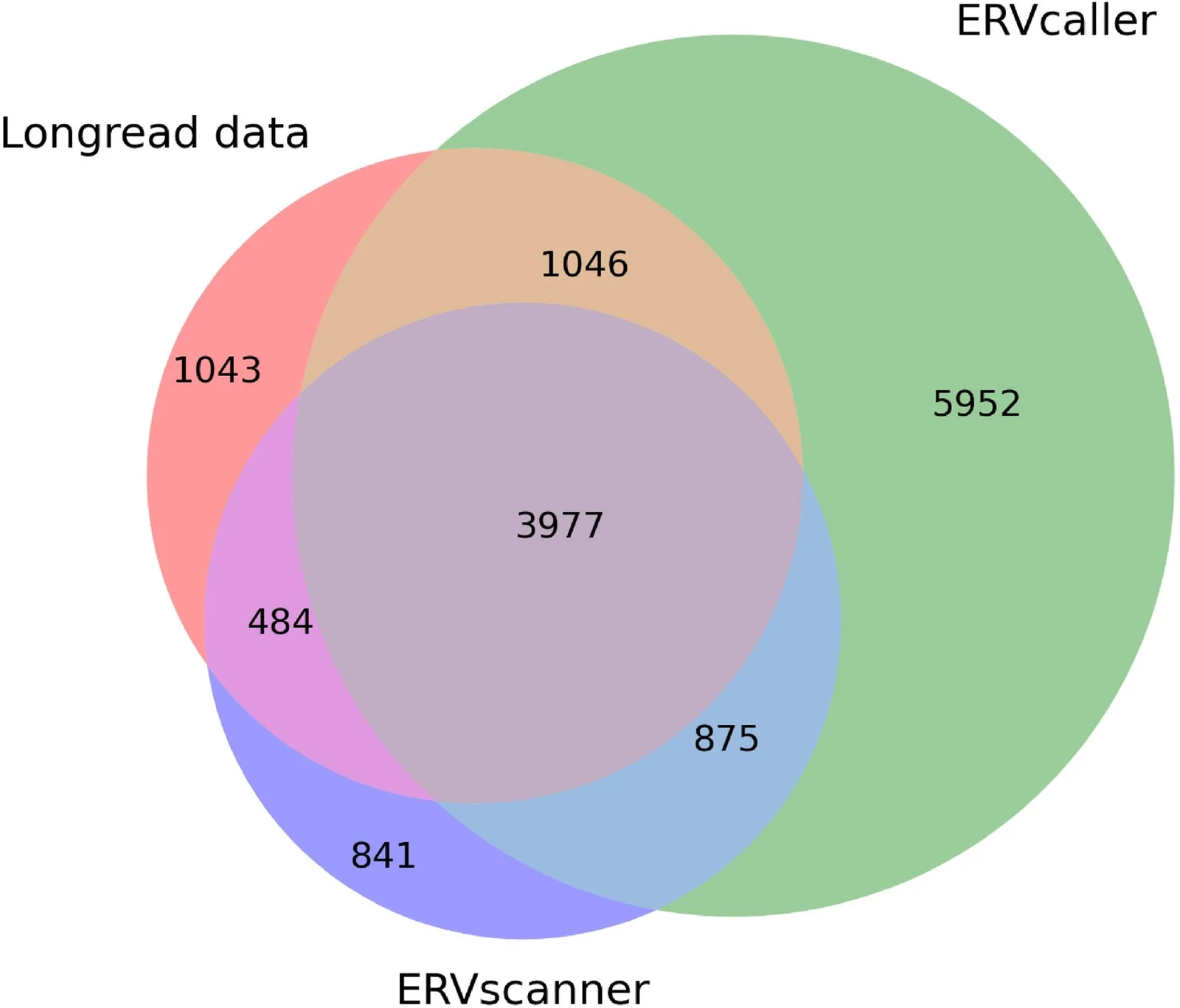
Venn diagram of detected nonreference ERV insertions in the JF1 strain genome. Long-read represents high-coverage PacBio data, while ERVcaller and ERVscanner utilized 30× short-read data.

The majority of mispredictions in ERVscanner are due to misclassification of the sequence class rather than incorrect insertion detection. When concordance was defined solely by positional overlap with any insertion of 200 bp or longer identified in the long-read sequencing data, 88.1% of the ERVscanner calls were supported by the long-read data.

We also evaluated data from 11 inbred strains for which both short- and long-read sequencing data were available (Ferraj et al. 2023). The summary of concordance rates is shown in Table 1. In general, more insertions were detected in the long-read data than by ERVscanner using short-read data. Approximately 32% to 37% of the long-read calls were supported by ERVscanner calls, while 76% to 82% of the ERVscanner calls were also detected in the long-read data.

**Table 1.** Numbers of ERV insertions identified in 11 inbred strains.

Strain	Long-read call	ERVscanner call	Both	Long-read CR^a^	ERVscanncer CR^a^
129S1	5,478	2,541	2,022	0.37	0.80
AJ	5,260	2,390	1,938	0.37	0.81
BALBcByJ	4,890	2,168	1,793	0.37	0.83
BALBcJ	4,871	2,212	1,807	0.37	0.82
C3HHeJ	5,406	2,470	1,995	0.37	0.81
CAST	15,253	6,504	5,114	0.34	0.79
DBA	5,497	2,474	1,991	0.36	0.80
NOD	5,563	2,528	2,004	0.36	0.79
NZO	5,537	2,481	1,992	0.36	0.80
PWK	16,054	6,714	5,135	0.32	0.76
WSB	8,046	3,604	2,881	0.36	0.80

^a^CR: concordance rate: fraction of insertions supported by the other dataset.

We further calculated genetic distances among inbred strains using the information on insertion loci shared between strains. Both datasets resulted in identical tree topologies with highly similar branch lengths (Supplementary Fig. 4).

### Population genomic search for ERV across wild house mouse genomes

We analyzed short-read mapping data from 163 wild house mice using ERVscanner (Supplementary Table 2), and identified an average of 7,103, 3,524, and 6,996 nonreference ERV insertions on autosomes per individual in the subspecies *castaneus*, *domesticus*, and *musculus*, respectively. The output VCF file is available at Zenodo (doi: 10.5281/zenodo.22019831). The distribution of the insertions for each individual is presented in Fig. 2. As expected, the subspecies *domesticus* harbored the fewest ERV insertions, because the reference genome is primarily derived from the subspecies *domesticus*. We found that the class composition of ERV insertions differs among subspecies, with significant differences detected for each ERV class (Kruskal–Wallis test; class I: *P* = 1.21 × 10^−24^; class II: *P* = 9.53 × 10^−15^; class III: *P* = 3.54 × 10^−23^). Further pairwise tests suggested that ssp. *musculus* tends to harbor more class I and class II insertions and fewer class III insertions, compared with ssp. *castaneus* and *domesticus* (all adjusted *P* < × 10^−7^; Dunn's test after Holm correction).

**Fig. 2.**
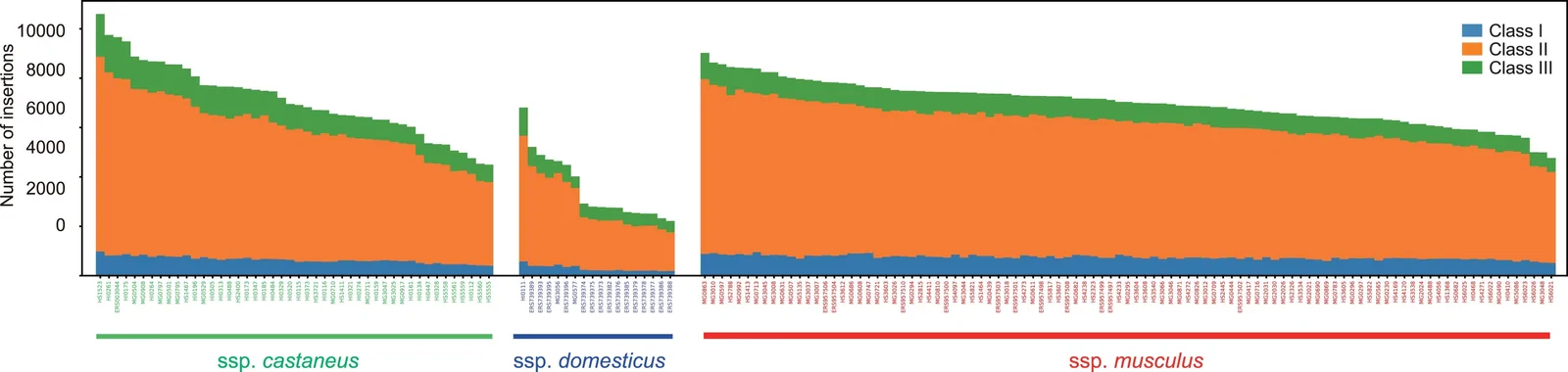
Histogram of loci with nonreference ERV insertions among all wild house mouse individuals. The labels of subspecies were determined by the majority of genomic components inferred by ADMIXTURE software (Fujiwara *et al*. 2024).

We asked whether global population structures could be reconstructed using the pattern of nonreference ERV insertions. PCA was performed using ERV insertions as markers, and the results reflected the subspecies population structures of wild house mice (Supplementary Fig. 5).

### Functional effect of ERV insertions

In total, we identified 178,334 insertion loci that were not present in the reference genome among 163 wild house mouse genomes. As previously reported (Zhang et al. 2008), there was a strand bias of ERV insertions in intronic regions. We observed 62,720 insertion loci overlapping with the transcribed regions, and among them, 41,191 ERVs (66%) were inserted in a reverse orientation to the transcription.

We further investigated the relationship between integration sites and local recombination rates. We first calculated average local recombination rates and number of insertions for each 20kbp-length window and computed Spearman correlation coefficient (*ρ*), but did not observe a significant correlation (*ρ* = 0.0026, *P* = 0.45). We next investigated the association between insertion allele frequency and local recombination rate, and found a statistically significant but weak negative correlation, suggesting that common insertions preferentially reside in low-recombination regions (*ρ* = −0.0158; *P* = 7.20 × 10^−11^; Supplementary Fig. 6).

We identified 320 genes with 325 ERV insertions disrupting protein-coding regions (Supplementary Table 3). Functional enrichment analysis using Metascape revealed significant enrichment of several gene clusters (Supplementary Fig. 7). For example, genes associated with response to chemical stimulus (GO:0001580), including 64 olfactory receptor (OR) genes and 14 vomeronasal receptor (*Vmnr*) genes, were significantly enriched. Other overrepresented categories included metabolism of small nitrogenous compounds (mmu00410; WP522) and immune system-related processes (GO:0002218), such as macrophage migration, monocyte chemotaxis, and leukotriene metabolism.

### Polymorphic insertion of restriction factor *Fv4*

To examine the causal relationship between ERV insertions and natural selection, we focused on the *Fv4* gene, a major restriction factor for MLV infection that is absent from the current reference genome. Long-read sequencing data revealed that the JF1 mouse strain possesses *Fv4* at chr12:80,797,701 in the GRCm38 reference genome. ERVscanner successfully inferred that *Fv4* had presence/absence polymorphism in wild house mouse individuals at the same position. The geographic distribution of *Fv4* (Supplementary Fig. 8) was consistent with the findings of Boso et al. (2021) but offers higher geographic resolution. In general, East Asian house mice exhibit a *musculus*-like genetic background in northern China and a *castaneus*-like background in southern China, with the Yangtze River (or Qinling Mountains) serving as an approximate boundary. *Fv4* is primarily restricted to the subspecies *castaneus*, but some East Asian *musculus*-like individuals along the eastern coastal regions, including northern China (Shandong and Hebei Provinces), Korea, Japan, and Russian Primorye, also harbor *Fv4*. Among these, Japanese and Russian samples show admixed subspecies origins. In contrast, 7 individuals from northeastern China, which have a strongly *musculus*-like genetic background, do not harbor *Fv4*.

The distribution pattern of *Fv4* suggests that the gene may have introgressed from the subspecies *castaneus* to *musculus* during secondary contact in East Asia. We computed local *f*_4_ statistics using the configuration *f*_4_(SPR, S-CHN; KOR, NE-CHN), which becomes negative if introgression occurred from the S-CHN population into the Korean population (KOR), but not into the northeastern Chinese population (NE-CHN), with *M. spretus* (SPR) as the outgroup. Because the S-CHN population is mostly composed of subspecies *castaneus* ancestry, whereas the N-CHN and KOR populations are mostly composed of subspecies *musculus* ancestry, the *f*_4_ configuration tests whether KOR shows excess local genetic affinity to S-CHN relative to N-CHN. As shown in Fig. 3, the *f*_4_ statistics were strongly skewed toward negative values around the *Fv4* insertion sites, indicating that the genomic segments surrounding the *Fv4* insertions were introgressed into the Korean population but not into the northeastern Chinese population. Using RFMIX2, we also confirmed that haplotypes surrounding the insertion site contained genomic fragments derived from the southern Chinese population (Supplementary Fig. 9).

**Fig. 3.**
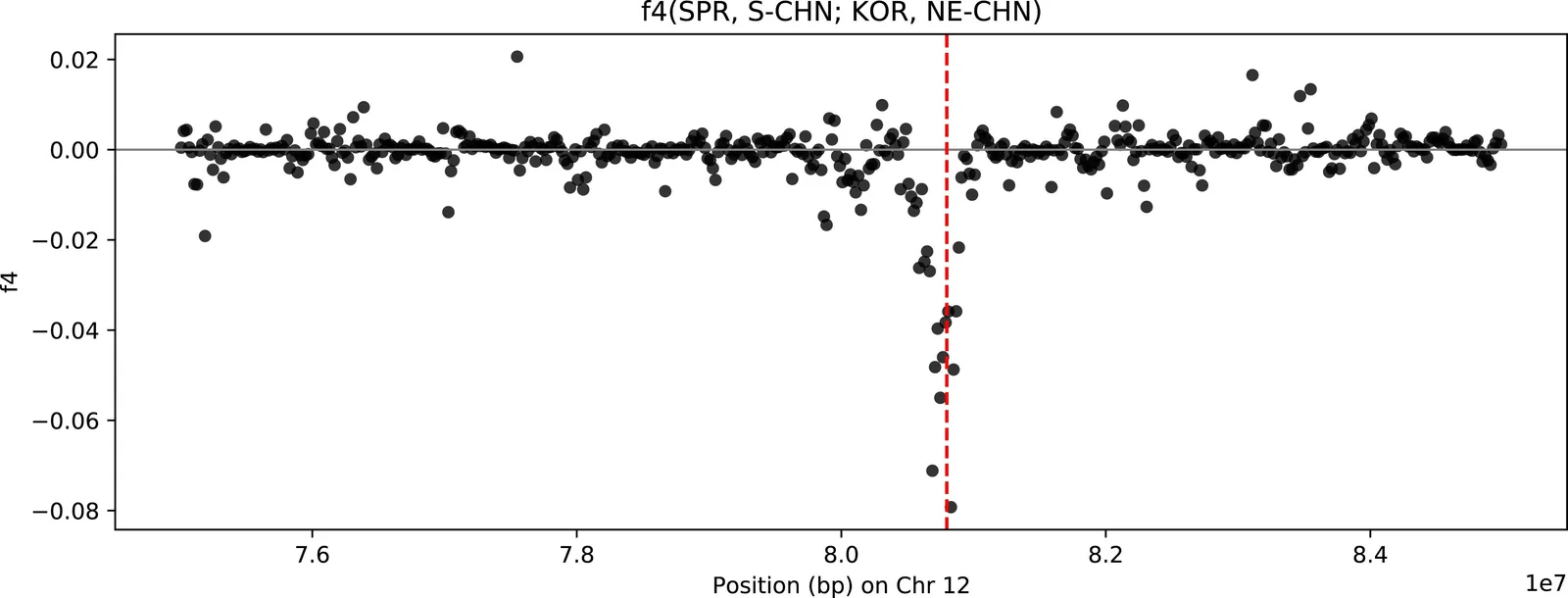
*F*_4_ statistics were calculated using SNPs in 20-kb windows around the integration site of *Fv4*. The dashed vertical line represents the inferred integration site of *Fv4*. The *f*_4_ statistics were calculated under the configuration *f*_4_(SPR, S-CHN; KOR, NE-CHN), where SPR, S-CHN, KOR, and NE-CHN represent *M. spretus*, southern Chinese *M. musculus castaneus*, Korean *M. m. musculus*, and northeastern Chinese *M. m. musculus*, respectively. Assuming that *Fv4* originated from *M. m. castaneus*, negative *f*_4_ values indicate introgression from S-CHN into KOR, but not into NE-CHN.

To further investigate whether the spread of *Fv4* has been driven by adaptive evolution, we conducted a genomic scan for positive selection in Korean samples. We plotted nucleotide diversity and Tajima's *D* statistics surrounding the *Fv4* insertion site (Fig. 4) using SNP data. Additionally, using northeastern Chinese samples, which do not possess *Fv4*, as a reference, we calculated and plotted *F*_ST_ and XP-nSLs (Fig. 4). The results indicate a reduction in polymorphisms, an excess of rare alleles, high differentiation between populations, and homogeneous haplotype structures, all signatures consistent with a selective sweep. We confirmed that excluding windows overlapping the *Fv*4 insertion site and its surrounding 500-bp region did not substantially change the observed patterns. The 20-kb window centered on the *Fv4* insertion site contained a site with an XP-nSL score in the top 0.01% on chromosome 12, and the overall distribution of XP-nSL scores within the window was significantly shifted toward higher values (Mann–Whitney U test, *P* below the numerical precision returned by SciPy; Supplementary Fig. 10). In addition, 2% of sites within the window had XP-nSL scores in the top 0.1% of scores across chromosome 12. These findings support the hypothesis that *Fv4* has undergone adaptive introgression from the subspecies *castaneus* to the subspecies *musculus* in East Asia, likely facilitated by secondary contact between these subspecies. The presence of *Fv4* in Korean *musculus*-like populations, despite their genetic background, suggests that positive selection has favored the retention of this gene, potentially due to its role in conferring resistance to MLVs.

**Fig. 4.**
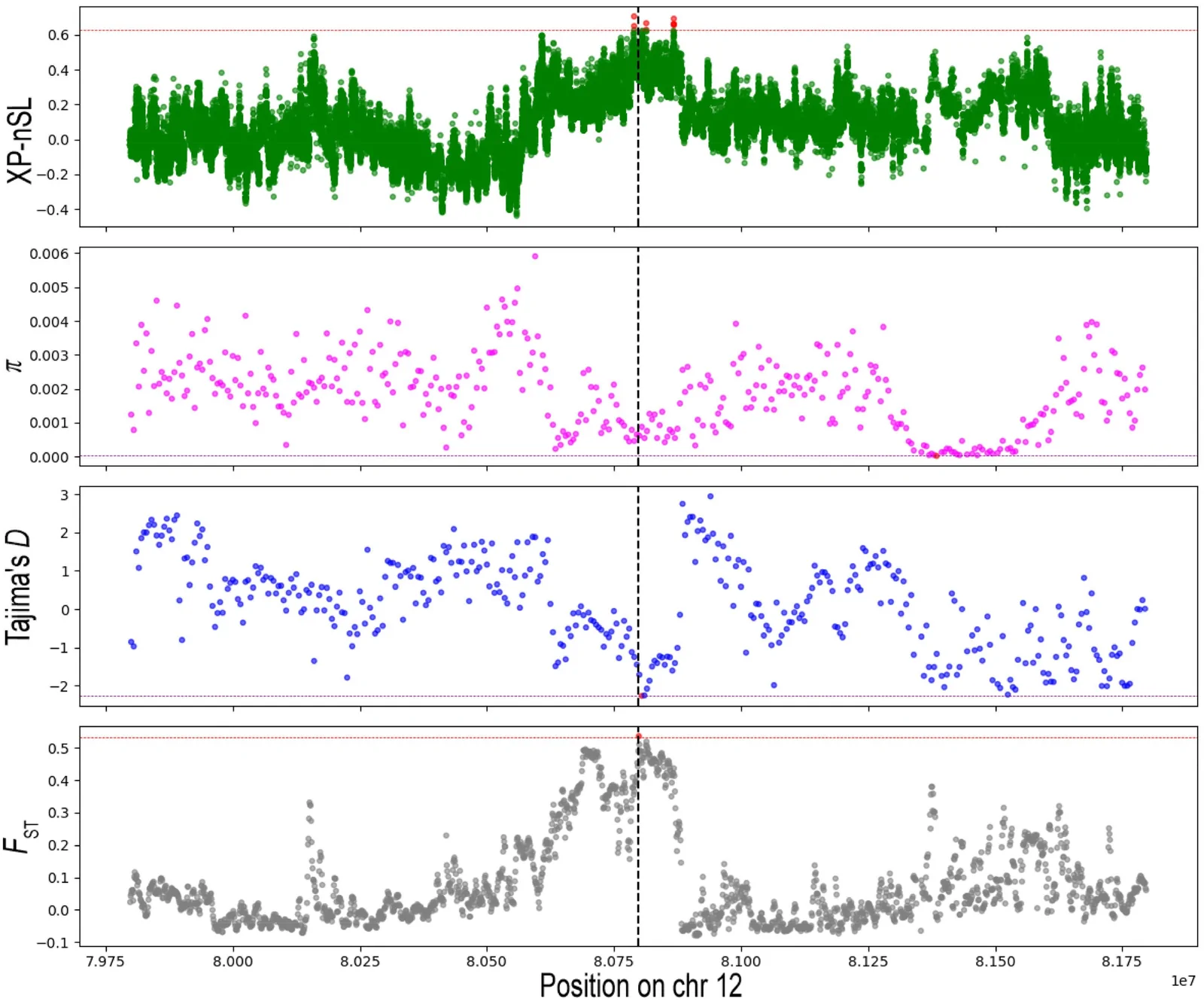
Genomic signature of selective sweep around the insertion of *Fv4* in the Korean population. The vertical dashed line in the center of the plot represents the estimated insertion site of *Fv4*. Horisontal dashed lines indicate the thresholds for the top 0.01% and bottom 0.01% of the chromosome-wide window distribution. For the XP-nSL and *F*_ST_ calculation, northeastern Chinese individuals, which also possess strong *musculus*-like genetic background, were used as the control population.

We also inferred the timing and strength of selection using genealogy-based methods implemented in the RELATE and CLUES software (Speidel et al. 2019; Stern et al. 2019). We inferred the past demography of the subspecies *musculus* and adjusted the branch length using the inferred demography (Supplementary Fig. 11). After the correction, we estimated that the increase of *Fv4* insertion frequency began approximately 1,000 generations ago, with a selection coefficient of 0.47%. Log-likelihood ratio against the neutral model was 8.50. The trajectory of *Fv4* insertions over time in the subspecies *musculus* is shown in Supplementary Fig. 12. The effective population size (*N*_e_) of the subspecies *musculus* has been estimated to be approximately 4 × 10^4^ after 10,000 years ago, and 1,000 generations correspond to approximately 0.0125 units of 2*N*_e_ generations, indicating that the inferred selection occurred on a very recent evolutionary timeframe.

### Candidate ERV insertions associated with selective sweep signals

To identify conservative candidates for ERV insertions associated with selective sweep signals, we focused on the strongest haplotype-based selection signals in the Korean population. We focused on this population because it showed a relatively homogeneous population structure and had a sufficient sample size for haplotype-based analysis. We selected SNPs in the top 0.1% of nSL scores and searched for ERV insertions located near these SNPs, excluding regions close to exons to reduce the possibility that the signal was driven by nearby protein-coding genes. This filtering retained 65 candidate sweep regions that were located near ERV insertions but not near exons (see Methods). To prioritize ERV insertions that could plausibly be associated with recent sweep signals, we examined the allele frequencies of the nearby insertions in the Korean population and in the subspecies *castaneus*. Under a scenario in which an ERV insertion contributed to a recent sweep in the Korean population, the insertion would be expected to be at relatively high frequency in Korea but low frequency in the subspecies *castaneus*. Based on this criterion, we identified 3 candidate insertions with frequencies >0.5 in the Korean population and higher frequencies than in the subspecies *castaneus*. In all 3 cases, the corresponding insertion was also present in the JF1 mouse strain, allowing us to infer the insertion sequence using available long-read sequencing data. These insertions were treated as prioritized candidates for sequence characterization, rather than as definitive causal variants.

Table 2 presents the candidate insertions. Among these, 1 locus (chr1:3723618) is a solo LTR derived from an IAP element; another (chr7:42156902) is a nearly full-length RLTR10 insertion lacking intact protein-coding capacity; and a third (chr18:21435569) encodes a 1,153-amino-acid protein in the longest open reading frame, corresponding to ERV gag and pol proteins.

**Table 2.** ERV insertions located near selective sweep signals in the Korean population.

Position	Class	Name	Length	Freq. Korea	Freq. *castaneus*
chr1:3723618	ERV2	IAPLTR1a_Mm	764	0.89	0
chr7:42156902	ERV2	RLTR10	2,685	0.94	0.08
chr18:21435569	ERV1	RLTR6B_Mm	3,387	0.76	0.17

## Discussion

### Evaluation of methods

In this study, we conducted a comprehensive search for nonreference ERV insertions in wild house mouse genomes and identified 178,334 potentially polymorphic insertion loci across 163 individuals. To facilitate the analysis, we developed a scalable pipeline for identifying nonreference ERV sequences designed to efficiently handle large datasets. Our pipeline is optimized for population genomic data and leverages shared insertions among individuals to enhance detection accuracy.

Although our benchmarking was not intended to provide a comprehensive comparison of all ERV insertion detection algorithms, the analyses using the JF1 mouse and 11 additional inbred strains indicate that ERVscanner can recover a substantial fraction of long-read-supported ERV insertions and assign their ERV classes with high concordance (Table 1).

One limitation of our benchmarking is that we used long-read-based calls as a reference set, although structural-variant detection is highly dependent on datasets and analytical methods. Further simulation studies that mimic actual transposable element insertions would be necessary for a rigorous evaluation of detection performance.

The total number of insertions identified using 163 wild house mouse genomes exceeds that reported in recent long-read sequencing studies based on a limited number of inbred strains (Ferraj et al. 2023). The observed difference is expected, as the genetic diversity represented by currently used inbred strains accounts for only a small fraction of the total diversity within the species (Yang et al. 2011). Overall, our results suggest that ERV insertions make a substantial contribution to the genetic diversity of wild house mice.

### Genomic pattern of ERV insertions

Multiple genomic factors may contribute to the distribution patterns of ERVs across genomes. ERVs and other transposable elements tend to integrate into regions of open chromatin, while unequal crossing-over may facilitate their removal (Campos-Sánchez et al. 2016; Kent et al. 2017). Additionally, some ERVs contain sites that promote recombination (Edelmann et al. 1989), whereas ERV integration into functional genomic regions can negatively impact fitness and may be selectively removed from the genome (Campos-Sánchez et al. 2016). These opposing forces make it difficult to predict whether ERVs are preferentially found in high or low-recombination rates.

Our data focusing on polymorphic ERVs did not reveal an association between the density of ERV insertions and local recombination rate. In contrast, we observed a significant negative correlation between the allele frequency of ERV insertions and recombination rate, such that insertions tend to be at lower frequency in regions of high recombination.

The observed pattern is consistent with the possibility that purifying selection against ERV insertions is more efficient in high-recombination regions, where linkage effects are reduced. In addition, recombination-associated processes, such as deletion via unequal crossing-over, may further decrease the persistence of ERV insertions in these regions. These mechanisms could lead to an excess of low-frequency insertions in high-recombination regions, even if the overall insertion density shows little association with recombination rate.

### Genes affected by ERV insertions

Several observations suggest that polymorphic ERV insertions may have functional consequences. The overrepresentation of OR genes likely reflects relaxed purifying selection acting on this large and partially pseudogenized gene family, highlighting how insertion tolerance varies across the genome (Zhang and Firestein 2002). In addition, enrichment of genes involved in metabolic pathways raises the possibility that ERV insertions contribute to standing functional variation in wild populations. In addition to the directly disrupt genes, ERVs may also modify gene regulation. The ERVB4 insertion within *Ednrb*, which underlies the coat color phenotype of JF1 mice (Tanave and Koide 2020), illustrates how such events can have pronounced phenotypic effects.

### ERV insertions and adaptation

While ERV insertions can influence organismal phenotypes in various ways, an intriguing hypothesis is that ERVs may act as restriction factors against similar exogenous retroviruses (Goff 2004). However, molecular evidence for this mechanism remains scarce. In this study, we searched for retroviral-derived restriction factors not present in the reference genome, *Fv4*, *RMCF1*, and *RMCF2*, but only *Fv4* was detected by our pipeline. *Fv4*, a restriction factor that enhances resistance to MLVs but is absent from the mouse reference genome, serves as an ideal model for studying the evolutionary dynamics of ERV insertions in mammalian immunity (Boso et al. 2021). Derived from MLVs, *Fv4* retains only the *env* gene in complete form, which encodes an envelope protein with several substitutions and blocks MLV receptors, thereby inhibiting infection. The gene likely originated in the common ancestor of the subspecies *castaneus*, but previous studies have identified *Fv4* in some individuals of the subspecies *musculus* (Inaguma et al. 1991; Boso et al. 2021).

Our global population-wide survey revealed that individuals from northern China, Korea, Japan, and Russian Primorye carry *Fv4*. Notably, the northern Chinese and Korean samples predominantly exhibit *musculus*-like genetic backgrounds, suggesting that *Fv4* spread in these populations despite restricted gene flow, likely due to its strong selective advantage. Population genetic analyses, using various summary statistics, detected multiple signatures of selective sweeps around *Fv4* insertion site, supporting a scenario of adaptive introgression from the subspecies *castaneus* to *musculus* in this region. Genealogical analysis with RELATE and CLUES software estimates the spread occurred approximately 1,000 generations ago. Notably, the signature of selection was detected using SNP data alone. However, without investigating polymorphic ERV insertions, *Fv*4 would not have been identified as a candidate driver of the strong selective sweep.

Although the Korean sample size was modest (Ma et al. 2015), the analysis was focused on a strong localized signal around *Fv*4 rather than on an exhaustive genome-wide search for weak selection signals. We therefore considered the analysis with this sample size informative for this focused analysis, while recognizing that it limits the precision of allele-frequency estimates and the power to detect weaker or more complex signals.

A previous study has also revealed the adaptive introgression of another well-studied restriction factor, *Fv1* in the Japanese population (Fujiwara et al. 2024). *Fv1*, encoding a gag protein derived from MuERV-L, is present in the reference genome, but has 2 distinct alleles, B and N, with different protein lengths showing restriction to different types of MLVs. The distribution of N and B alleles is characteristic among subspecies, and Fujiwara et al. (2024) suggested that the *castaneus*-type B allele has been adaptively introgressed into the Japanese population. The examples of *Fv1* and *Fv4* indicate that the adaptive introgression of ERV-derived restriction factors plays a key role in the evolution of genomes under secondary contact of genetically differentiated populations.

Here, we identified 3 candidate ERV insertions that may have contributed to selective sweeps in the Korean population. Although we lack direct experimental evidence for the functional and selective effects of the ERV insertions shown in Table 2, the corresponding insertions were also detected in the JF1 mouse strain using long-read sequencing data in all 3 cases. This provides independent support that these candidate loci represent true ERV insertions rather than short-read-specific false positives. We therefore treated these insertions as prioritized candidates for sequence characterization, which may serve as valuable resources for studying the relationship between ERV integrations and adaptive evolution.

## Conclusions

The findings in this study highlight ERVs as a source of genetic diversity with the potential to influence host adaptation. By providing both an adaptive introgression event (*Fv4*) and a set of prioritized candidate loci, our study generates testable hypotheses regarding the evolutionary consequences of ERV insertions. More broadly, ERVscanner establishes a practical approach for detecting such polymorphisms in short-read datasets, enabling scalable discovery in systems where long-read sequencing remains limited. The catalog and framework presented here offer a foundation for both comparative studies across mouse populations and extension to other species, promoting the integration of ERV dynamics into mainstream population and functional genomics.

## Supplementary Material

jkag270_Supplementary_Data

## Data Availability

All nucleotide sequence data used in this study were obtained from public databases. The accession numbers are listed in Supplementary Table 2. The ERVscanner code is available on GitHub (https://github.com/Qtom-99/ERVscanner). The list of ERV insertions among 163 wild house mice is available at Zenodo (DOI: 10.5281/zenodo.22019831) in VCF file format. Supplemental material available at G3 online.
