# Supplementary material for: Evolutionary dynamics of polymorphic endogenous retrovirus insertions across wild house mouse populations
Source: G3 (Bethesda). 2026 Sep 22;16(11):jkag270. doi: 10.1093/g3journal/jkag270 (PMC13732460; doi:10.1093/g3journal/jkag270)
Supplement: Supplementary file 1 [file jkag270_supplementary_data.zip › Supplemental_File_1_G3-2026-407241.pdf]

Supplementary File 1: Algorithm of ERVscanner

Overview

ERVscanner consists of four main processes: read filtering, identification of read clusters around insertions, filtering based on insertion content, and genotyping. A graphical representation is provided in Supplementary Fig. 1 and 2. Although ERVscanner is specifically designed for ERV sequences, it can be applied to other classes of mobile elements. The code of ERVscanner is available at Github (https://github.com/Qtom-99/ERVscanner).

The pipeline utilizes pre-defined ERV regions in the reference genome, as annotated by the Dfam database (Storer et al. 2021), which identifies these regions using a hidden Markov model. We refer to these annotated regions as masked regions (MRs). For further processing, ERVscanner requires BED-format files containing MRs and FASTA-format sequences of MRs. The MR sequences are reformatted to the sense (+) strand representation of ERVs, and consensus sequences of ERVs are included in the FASTA file in the preprocess step. Additionally, any missing ERV sequences that are not represented in the reference genome can be manually added to the FASTA file.

Read filtering

Using the BED file, ERVscanner first extracts potential read pairs supporting non-reference insertions. When a non-reference ERV insertion is present in a sample but absent from the reference genome, one read of a pair may map to the flanking genomic region around the insertion site, whereas the other read of the pair may map to a homologous ERV sequence elsewhere in the reference genome. We detect such discordant read pairs. Discordant pairs include those mapped to different scaffolds/chromosomes, mapped more than 10 kb apart in the reference genome, or mapped with incongruent orientations.

The extracted pairs are filtered based on MRs. When one read maps within an MR and the mate

maps outside an MR, only the read mapped outside the MR is retained if it has high mapping quality ($MQ \geq 30$). Read pairs in which both reads map within MRs are excluded from further analysis. Because detecting insertions within MRs is challenging with short-read data and prone to elevated false discovery rates, ERVscanner estimates insertions only from reads supported by high-confidence mappings outside MRs.

If reads were aligned using BWA-MEM or other ALT-aware mappers against non–telomere-to-telomere reference genomes, reads mapping to both primary and alternative/unplaced contigs may receive artificially high mapping quality scores on primary chromosomes. To reduce false positives, clusters composed of reads with secondary alignments to alternative/unplaced contigs were removed.

Identification of read clusters around insertions

After filtering, ERVscanner identifies clusters indicative of non-reference ERV insertions. This clustering step is based on the mapped positions, orientations, and read counts of the non-ERV-side reads in discordant read pairs, that is, the reads mapped to the flanking genomic regions rather than to ERV sequences. Here, forward and reverse refer to the strands to which the supporting reads are mapped on the reference genome. Non-reference insertions are expected to generate a forward and a reverse cluster near the insertion site. For each orientation, genomic intervals covered by supporting reads were first identified from the mapped read positions. Neighboring intervals separated by ≤200 bp were then merged into a cluster. Clusters supported by at least a predefined number of reads were defined as forward or reverse clusters. This threshold is adjustable and was set to five reads in this study. When a forward cluster and a reverse cluster occur within 200 bp of each other, the locus is classified as a candidate non-reference insertion locus. After loci were defined for each sample, loci within 50 bp were merged across all samples, generating a common list of insertion loci for subsequent steps.

Estimation of insertion contents

For each candidate locus, we estimated the insertion content by re-mapping reads paired with the cluster-supporting reads, using BWA against 361 non-redundant transposable element sequences in the genus *Mus* from Dfam (Supplementary Table S3). We further reassigned Long_Terminal_Repeat families to ERV1 and MaLR families to ERV3 according to previous studies (Stocking and Kozak 2008; Kawase and Ichiyanagi 2023). Hereafter, we refer to these library sequences as the Dfam NRPH (non-redundant profile HMM) consensus. The Dfam NRPH consensus represents consensus sequences of transposable elements after removing overlapping annotations.

Filtering loci based on insertion content

In this step, ERVscanner filters candidate loci based on the consistency between the flanking read clusters and the inferred insertion content. If the clusters correctly identify a non-reference insertion, the forward and reverse clusters are expected to be located on the left and right sides of the insertion site, respectively. Candidate loci in which the forward and reverse clusters did not correspond to the left and right sides of the insertion site, respectively, were discarded. As illustrated in Supplementary Fig. 2, we hereafter refer to these two sides of an inferred insertion site as the left and right sides.

For each candidate locus, ERVscanner uses the mapping results of reads paired with the cluster-supporting reads. To improve the accuracy of insertion-content assignment, these mapping results are aggregated across individuals under the assumption that polymorphic insertions at the same locus share the same insertion content. If the insertion content is correctly inferred, reads paired with the left and right supporting clusters are expected to map to the same library sequence with consistent orientations, as shown in Supplementary Fig. 2.

For each mapped library sequence, ERVscanner counts the numbers of reads derived from the left and right sides of the target sequence and uses the smaller value as the support score. This criterion requires support from both sides of the insertion. After calculating support scores for all candidate

library sequences, each candidate insertion is assigned to the library sequence with the highest support score. In this study, we used a minimum support score of three, although this threshold is adjustable in the settings. Candidate loci with support scores below this threshold are not retained as confidently annotated insertions.

Genotyping

For each valid cluster, ERVscanner records whether the insertion is present and optionally provides a provisional zygosity call in the output VCF file. Within each valid cluster, clipped reads are extracted using CIGAR information to identify precise breakpoints. For identifying the breakpoint, reads across samples with insertions are aggregated. If clipped reads from left and right clusters overlap around a breakpoint, the minimum position is assigned as the breakpoint to maintain consistency with the VCF format generated by general genotyping software.

Heterozygosity call is performed for each sample. Heterozygosity is inferred when at least three breakpoint-crossing reads cover a 30-bp window centered on the breakpoint, indicating support for the non-insertion allele. Valid clusters lacking a sufficient number of clipped reads are genotyped as “1/.” in the output VCF file, indicating the presence of an insertion but uncertainty regarding zygosity.

References

Kawase M, Ichiyanagi K. 2023. Mouse retrotransposons: sequence structure, evolutionary age, genomic distribution and function. Genes Genet. Syst. 98:337-351. https://doi.org/10.1266/ggs.23-00221.

Stocking C, Kozak CA. 2008. Endogenous retroviruses. Cell. Mol. Life Sci. 65:3383-3398. https://doi.org/10.1007/s00018-008-8497-0.

Storer J, Hubley R, Rosen J, Wheeler TJ, Smit AF. 2021. The Dfam community resource of transposable element families, sequence models, and genome annotations. Mob. DNA 12:2. https://doi.org/10.1186/s13100-020-00230-y.
