# Supplementary material for: Evolutionary dynamics of polymorphic endogenous retrovirus insertions across wild house mouse populations
Source: G3 (Bethesda). 2026 Sep 22;16(11):jkag270. doi: 10.1093/g3journal/jkag270 (PMC13732460; doi:10.1093/g3journal/jkag270)
Supplement: Supplementary file 1 [file jkag270_supplementary_data.zip › Supplementary_Figures_G3-2026-407241.pdf]

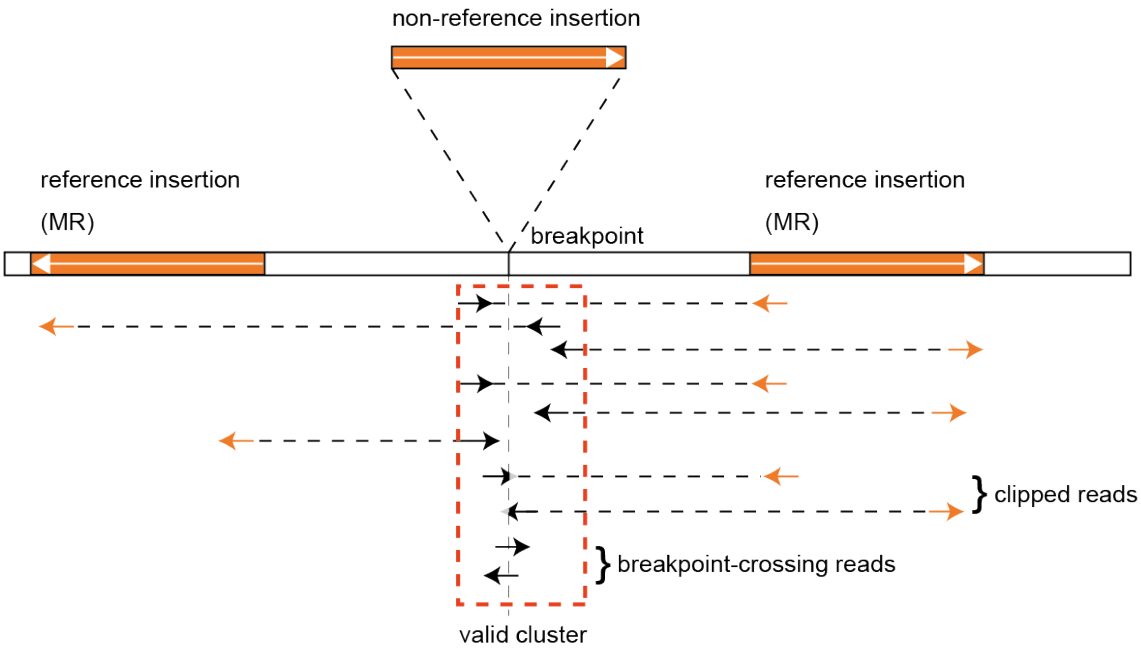

Supplementary Fig. S1

Schematic representation of the ERVscanner pipeline. Orange regions represent sequences homologous to ERVs. The topmost bar illustrates a non-reference ERV insertion to be detected. When DNA fragments spanning the insertion site are sequenced, discordant read pairs are generated in which at least one read maps to an ERV-derived masked region (MR) in the reference genome (indicated by orange arrows). The reads indicated by black arrows are referred to as left- and right-supporting reads that define the breakpoint cluster. Breakpoint positions are inferred from clipped reads, whereas breakpoint-crossing reads provide evidence for genotyping.

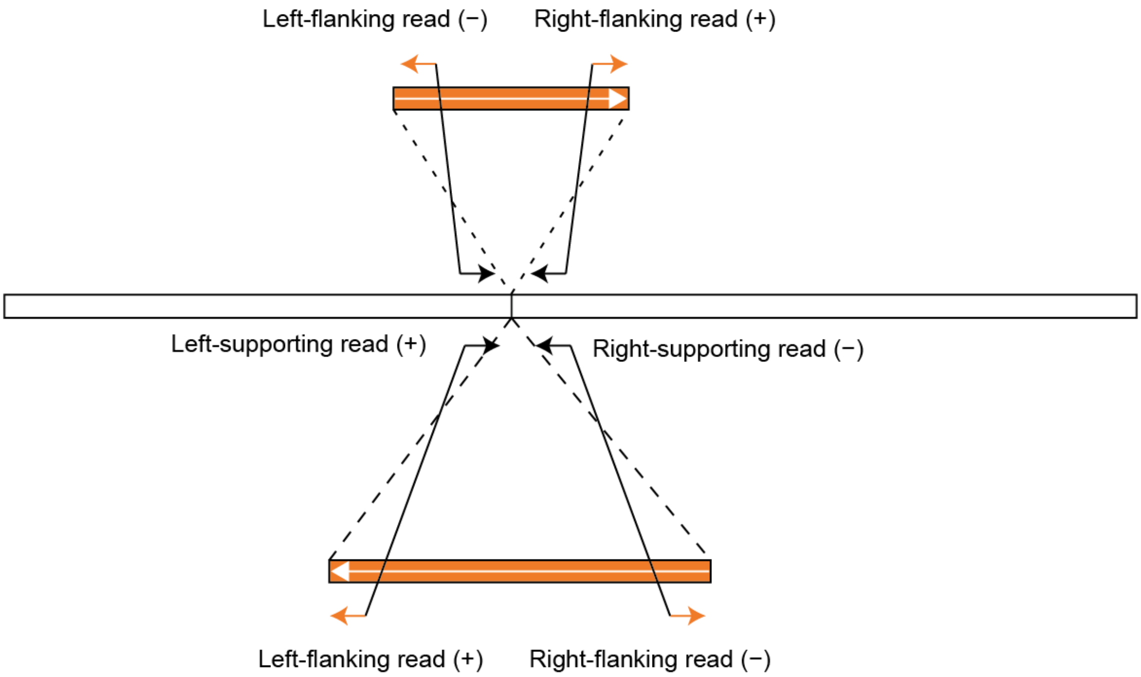

Supplementary Fig. S2

Schematic representation of how ERVscanner infers insertion contents. Reads mapped near the insertion site are referred to as supporting reads, and reads paired with the supporting reads are referred to as flanking reads. If the insertion content is correctly inferred, flanking reads from the left and right sides are expected to map to the target sequence with consistent orientations (left − and right +, or left + and right −).

For each target sequence, we counted the minimum number of left- and right-flanking reads with consistent orientations and defined this value as the support score. The target sequence with the highest support score was assigned as the inferred insertion content.

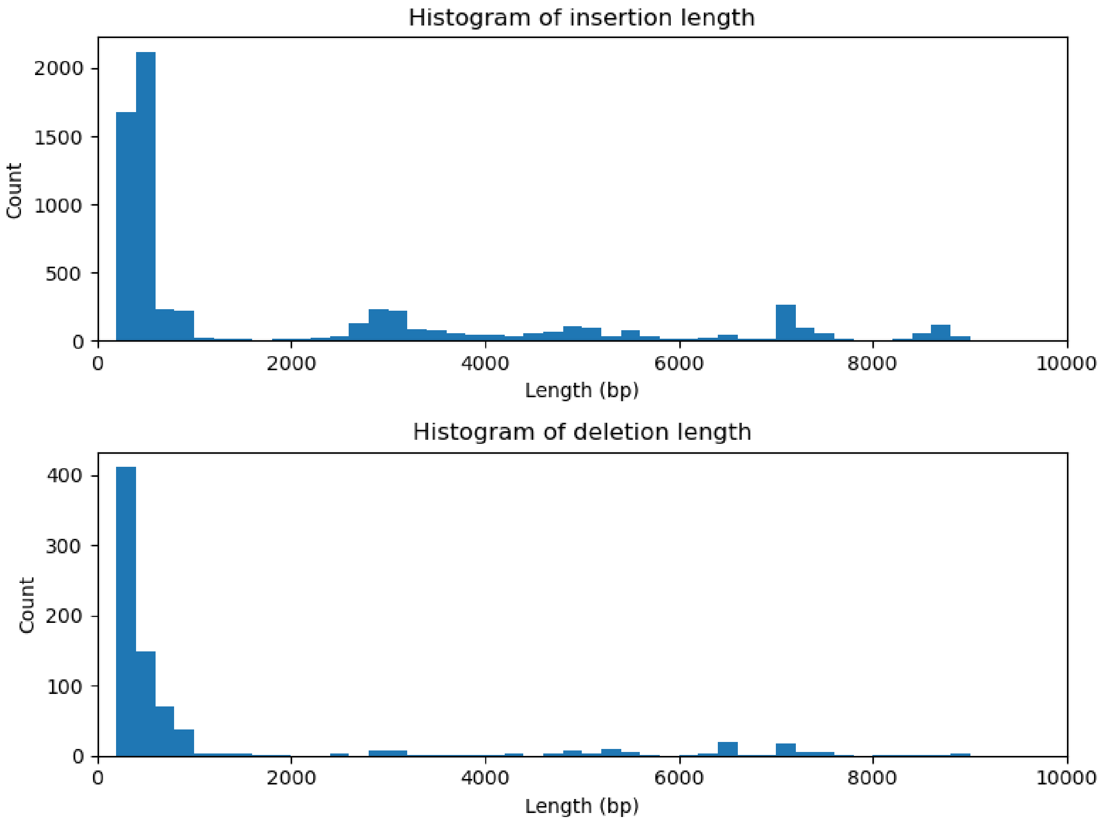

Supplementary Fig. S3

Distribution of insertion and deletions length of ERV variants in the JF1 genome, identified using a long-read sequencer.

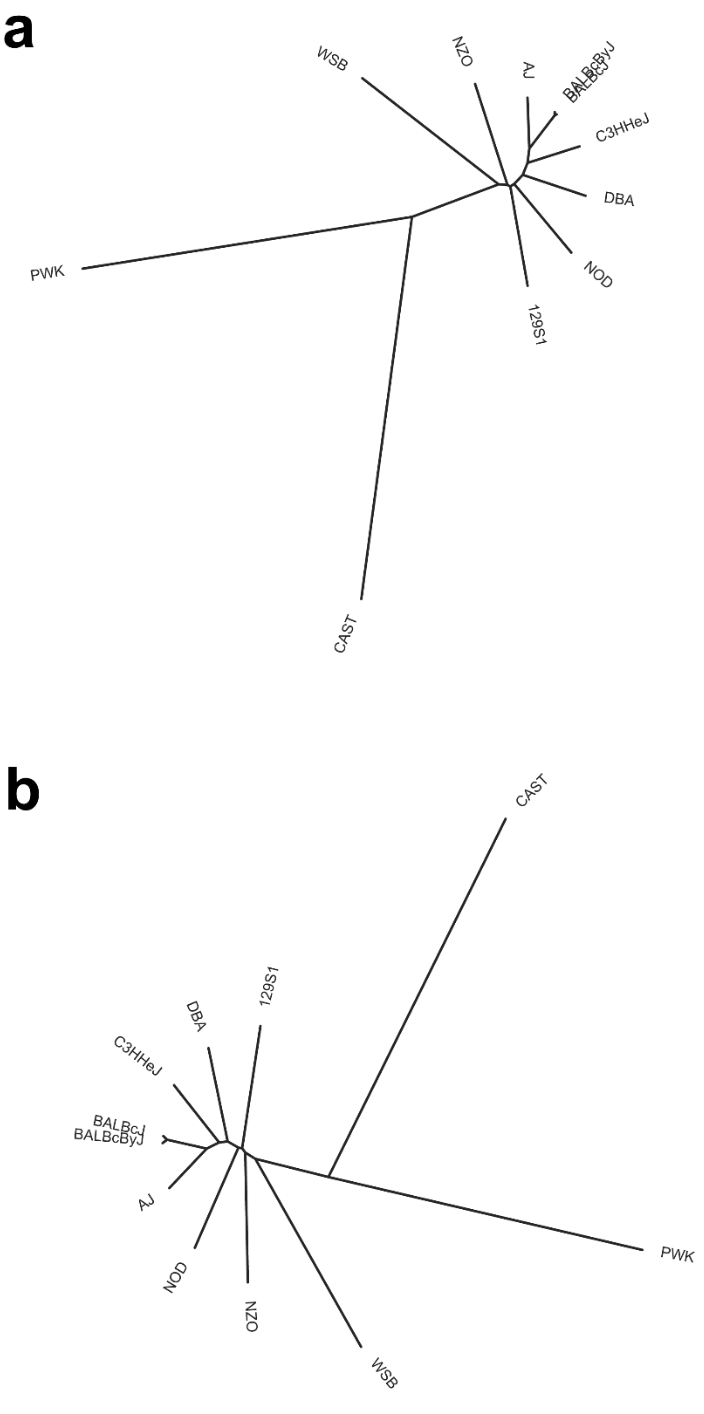

Supplementary Fig. S4

Neighbor-joining trees constructed using the numbers of shared insertions between strains. a) Long-read data by Ferraj et al. (2023). b) Short-read data analyzed using ERVscanner.

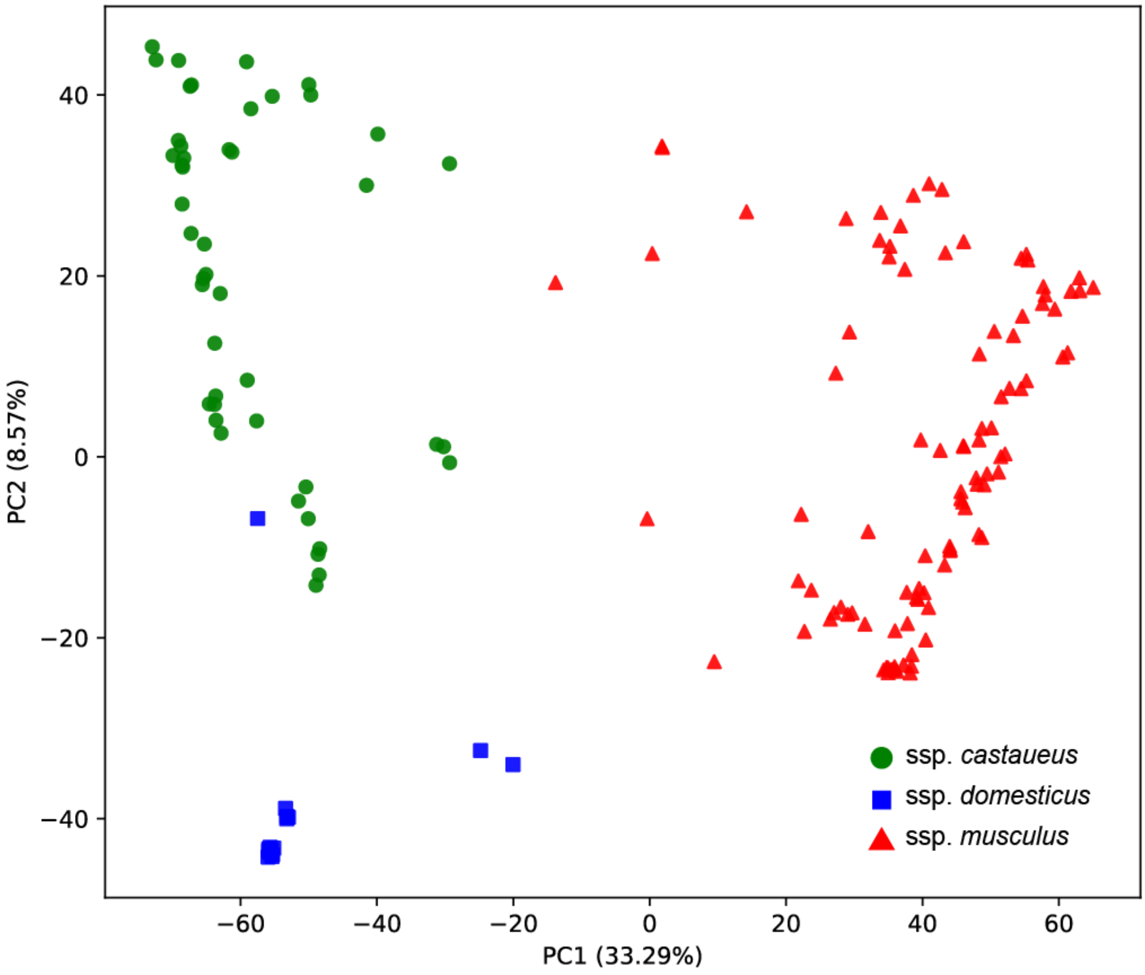

Supplementary Fig. S5

PCA plot for 163 wild house mice, using non-reference ERV insertions as genetic markers. PC1 and PC2 were plotted.

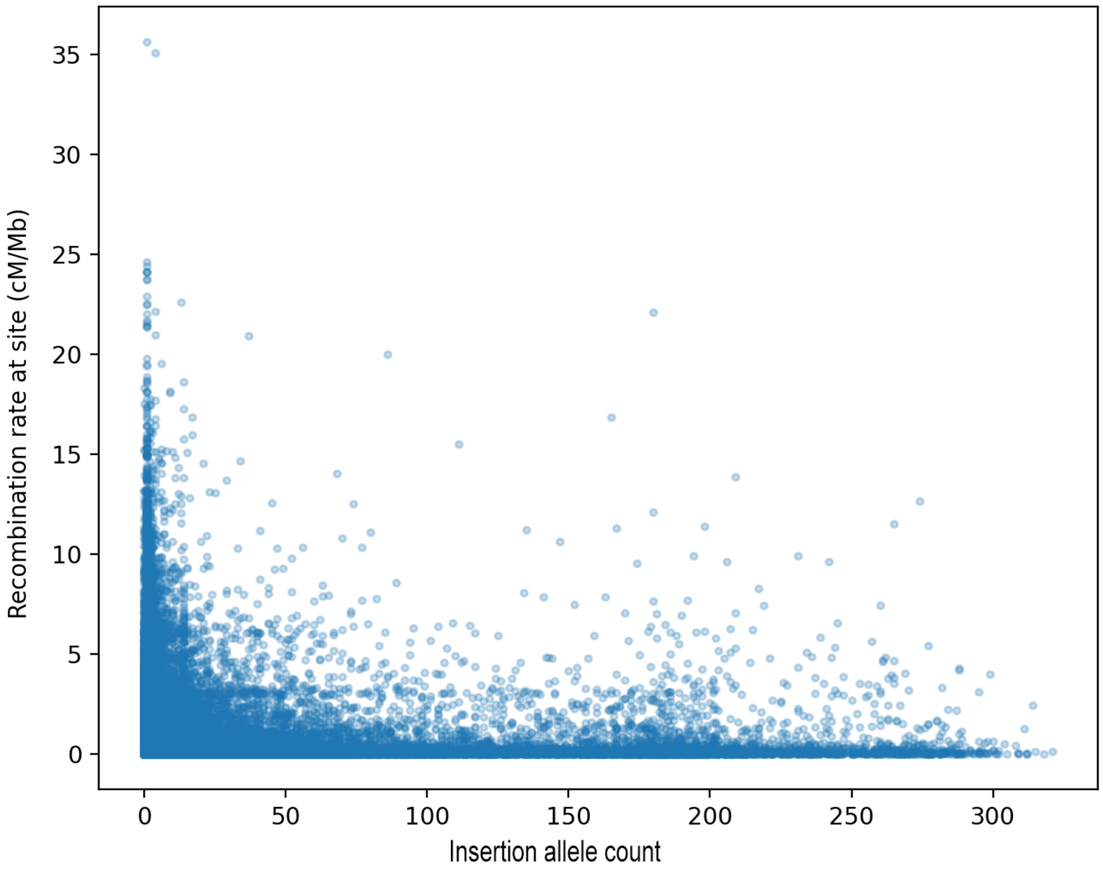

Supplementary Fig. S6

Scatter plot of allele count of non-reference insertions of ERVs and recombination rates at site.

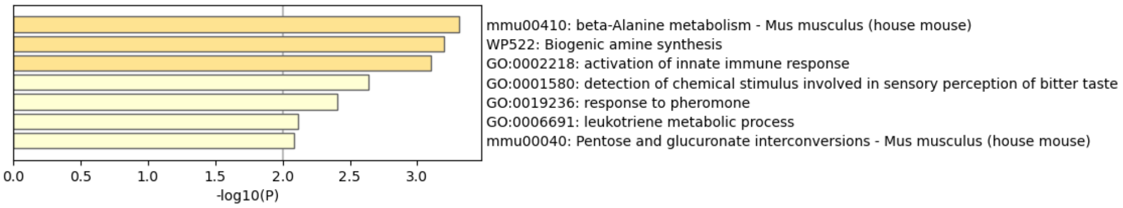

Supplementary Fig. S7

Enrichment analysis using Metascape. The enrichment of genes with ERV insertions in their protein-coding sequences is shown.

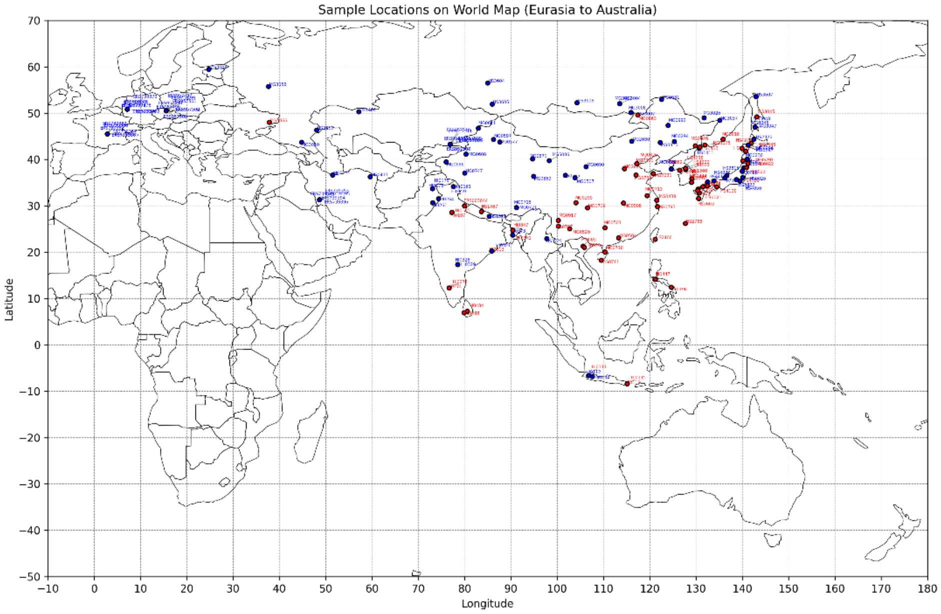

Supplementary Fig. S8

Distribution of *Fv4* insertions in Southern and Eastern Eurasia. The blue and red circles represent individuals with the absence and presence of *Fv4*, respectively.

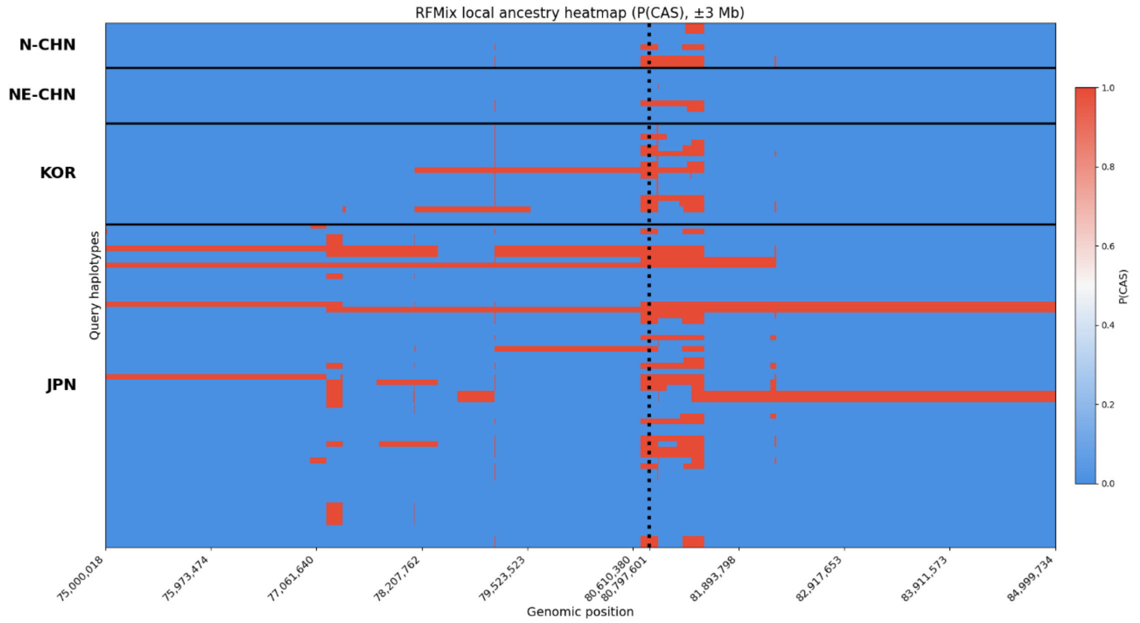

Supplementary Fig. S9

Local ancestry estimated using RFMIX2. The region of 6 Mbp around the insertion site of *Fv*4 is shown. The blue and red blocks represent the ancestry from subspecies *musculus* represented by Kazakhstan samples, and subspecies *castaneus* represented by southern Chinese samples. The horizontal dotted line indicates the insertion sites of *Fv*4.

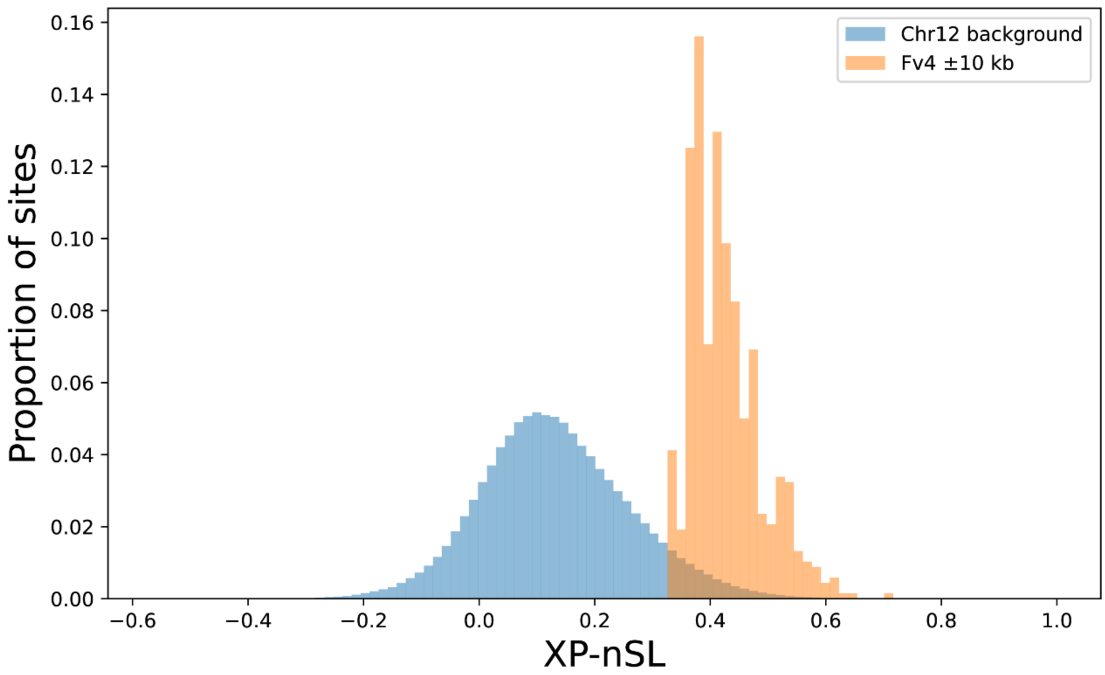

Supplementary Fig. S10

Site-level distributions of XP-nSL scores contrasting Korean and northern Chinese populations in the 20-kb window centered on *Fv4* and across chromosome 12.

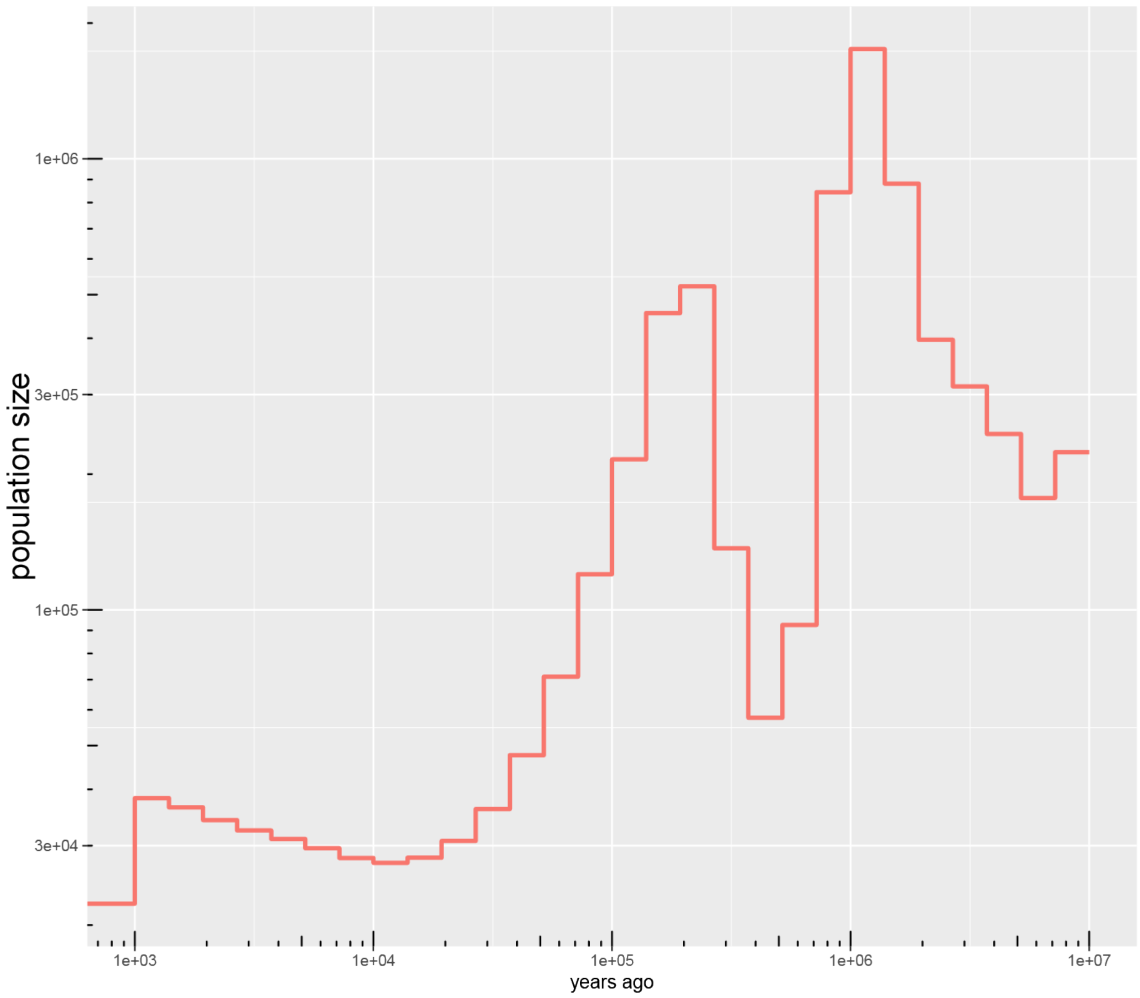

Supplementary Fig. S11

Trajectory of past effective population size of subspecies *musculus* using RELATE. Mutation rate of 5.7×10^{-9} per generation and 1 year of generation time were assumed.

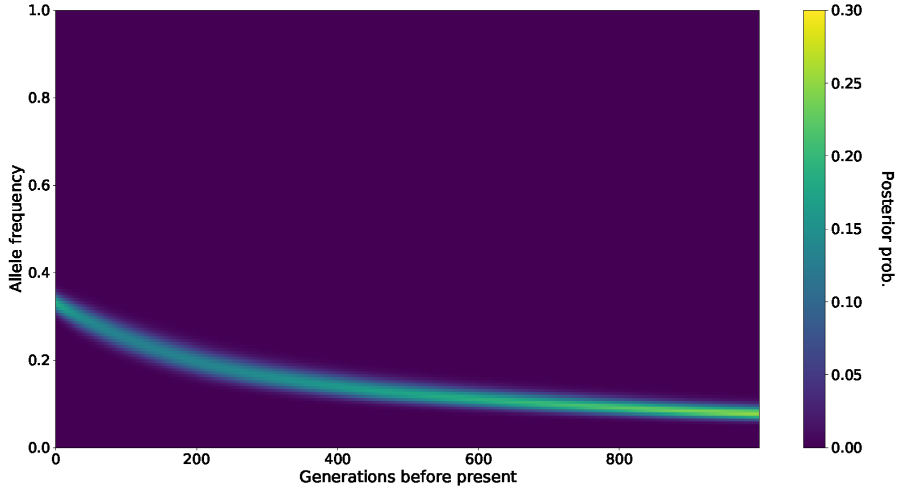

Supplementary Fig. S12

Estimated trajectory of *Fv4* insertions in the subspecies *musculus*. The genealogy around the *Fv4* insertion was estimated using RELATE, and the trajectory of allele frequency was inferred using CLUES.
